# Astrocytic Oxidative/Nitrosative Stress Contributes to Parkinson’s Disease Pathogenesis: The Dual Role of Reactive Astrocytes

**DOI:** 10.3390/antiox8080265

**Published:** 2019-08-01

**Authors:** Asha Rizor, Edward Pajarillo, James Johnson, Michael Aschner, Eunsook Lee

**Affiliations:** 1 Department of Pharmaceutical Sciences, College of Pharmacy Florida A&M University, Tallahassee, FL 32301, USA; 2 Department of Molecular Pharmacology, Albert Einstein College of Medicine Bronx, New York, NY 10461, USA

**Keywords:** astrocytes, oxidative stress, nitrosative stress, parkinson’s disease, neurological disorders

## Abstract

Parkinson’s disease (PD) is the second most common neurodegenerative disease worldwide; it is characterized by dopaminergic neurodegeneration in the substantia nigra pars compacta, but its etiology is not fully understood. Astrocytes, a class of glial cells in the central nervous system (CNS), provide critical structural and metabolic support to neurons, but growing evidence reveals that astrocytic oxidative and nitrosative stress contributes to PD pathogenesis. As astrocytes play a critical role in the production of antioxidants and the detoxification of reactive oxygen and nitrogen species (ROS/RNS), astrocytic oxidative/nitrosative stress has emerged as a critical mediator of the etiology of PD. Cellular stress and inflammation induce reactive astrogliosis, which initiates the production of astrocytic ROS/RNS and may lead to oxidative/nitrosative stress and PD pathogenesis. Although the cause of aberrant reactive astrogliosis is unknown, gene mutations and environmental toxicants may also contribute to astrocytic oxidative/nitrosative stress. In this review, we briefly discuss the physiological functions of astrocytes and the role of astrocytic oxidative/nitrosative stress in PD pathogenesis. Additionally, we examine the impact of PD-related genes such as α-synuclein, protein deglycase DJ-1( DJ-1), Parkin, and PTEN-induced kinase 1 (PINK1) on astrocytic function, and highlight the impact of environmental toxicants, such as 1-methyl-4-phenyl-1,2,3,6-tetrahydropyridine (MPTP), rotenone, manganese, and paraquat, on astrocytic oxidative/nitrosative stress in experimental models.

## 1. Introduction

Parkinson’s disease (PD) is the second most common neurodegenerative disorder and the most prevalent movement disorder worldwide [1]. PD is characterized by the progressive loss of dopaminergic neurons in the substantia nigra pars compacta (SNpc) and often the presence of cytoplasmic protein aggregates known as Lewy bodies, which result in decreased neurotransmission and motor deficits [2]. While genetic factors are known to directly contribute to ~10% of PD cases, the majority are idiopathic in nature [3,4]. The mechanisms of PD are not fully understood, but increasing evidence implicates astrocytic oxidative and nitrosative stress in PD pathogenesis [5].

The aging brain is uniquely vulnerable to oxidative and nitrosative damage [6], which can be seen in diverse neurodegenerative diseases such as PD, Alzheimer’s disease (AD), Lewy body dementia (LBD), and multiple system atrophy (MSA) [7,8,9]. The high consumption of glucose and oxygen in the central nervous system (CNS) requires continuous metabolic activity to satisfy energetic demands, and the reduction–oxidation (redox) reactions of metabolism produce reactive oxygen and nitrogen species (ROS/RNS) as natural byproducts [10]. Astrocytes, star-shaped glial cells, which surround neurons and closely associate with neuronal synapses, play a primary role in antioxidant production, detoxification of reactive species, and maintenance of redox balance in the brain [11].

**Figure 1:**
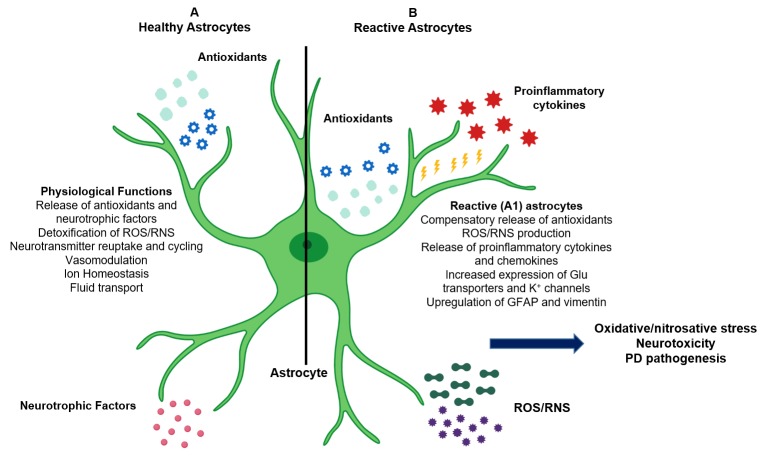
Role of astrocytes in the central nervous system (CNS) and in Parkinson’s disease (PD) pathogenesis. (**A**) Astrocytes provide structural and metabolic support to neurons, mediate neurotransmission and glutamate transport, and maintain ionic and vascular homeostasis. Astrocytes also secrete endogenous antioxidants (such as glutathione (GSH) and superoxide dismutase (SODs)) and neurotrophic factors into the extracellular microenvironment and are responsible for detoxification of reactive oxygen and nitrogen species (ROS/RNS) produced as byproducts of metabolism. (**B**) Reactive astrocytes produce additional antioxidants, proinflammatory cytokines and chemokines, and ROS/RNS. Chronic reactive astrogliosis leads to astrocytic oxidative/nitrosative stress, neuroinflammation, neuronal apoptosis, and PD pathogenesis.

In the healthy brain, astrocytes provide essential metabolic and functional support to neurons [11]. However, CNS injury or disease, the overproduction of ROS/RNS, or defects in detoxification can result in the activation of a coordinated astrocytic response: a series of biochemical and morphological changes collectively referred to as reactive astrogliosis [12] (Figure 1). Reactive astrocytes respond to acute cellular stress and work to limit CNS damage, but chronic astrogliosis can result in the sustained production of ROS/RNS and the release of proinflammatory molecules, which promotes neuronal injury and neurotoxicity [13]. Although this review highlights the role of reactive astrogliosis in PD and dopaminergic neurodegeneration, its effects are not solely confined to PD alone: reactive astrogliosis may also contribute to the pathogenesis of various neurological disorders including AD, dementia, and amyotrophic lateral sclerosis (ALS) [14].

Exposure to environmental toxicants such as 1-methyl-4-phenyl-1,2,3,6-tetrahydropyridine (MPTP), rotenone, and manganese (Mn) may contribute to the induction of reactive astrogliosis and consequent astrocytic oxidative/nitrosative stress [15,16,17]. Moreover, genes associated with PD pathogenesis, such as α-synuclein, protein deglycase DJ-1 (DJ-1), Parkin, and PTEN-induced kinase 1 (PINK1), regulate astrocytic function and may thus play an important role in mediating oxidative/nitrosative stress. Therefore, elucidating the role of astrocytic oxidative/nitrosative stress in PD pathogenesis may greatly expand our understanding of PD. In this review, we discuss the role of astrocytes in the etiology of PD, astrocytic oxidative/nitrosative stress as potential contributors to PD pathogenesis, and genes associated with astrocytic oxidative/nitrosative stress. Finally, we review the impact of environmental toxicants, such as MPTP, rotenone, Mn, and paraquat, on astrocytic oxidative/nitrosative stress and consequent neuropathogenesis.

## 2. The Role of Astrocytes in PD

### 2.1. Physiological Role of Astrocytes

Astrocytes are the most abundant glial cells in the CNS, where they account for 20–40% of total glia and are organized throughout the brain in non-overlapping domains [18]. There, they provide metabolic and structural support to neurons, mediate neurotransmission, maintain vascular and fluid homeostasis, and maintain ionic concentration in the mammalian brain [19]. Astrocytes closely interact with neurons through specialized projections known as astrocytic processes, which envelop neuronal cell bodies and synapses, contribute to the blood-brain barrier (BBB), and enable neuron–astrocyte cross-talk [12].

Although astrocytes are characterized by their unique star-shaped morphology and expression of glial fibrillary acidic protein (GFAP) or vimentin (Vim), two distinct subtypes, protoplasmic and fibrous astrocytes, carry out diverse functions across the CNS [20]. Protoplasmic astrocytes are the most prevalent subtype, and they are widely found in the gray matter of the brain [21]. The short, branching processes of protoplasmic astrocytes terminate in ‘astrocytic endfeet,’ which wrap around CNS blood vessels to form the outer BBB and regulate blood and fluid flow [22]. Protoplasmic astrocytes can also be found near synapses, where they provide essential structural and functional support to neurons [23]. The role of fibrous astrocytes is not well understood, but they are primarily located in white matter and have long, unbranched astrocytic processes, which make contact with cerebral vasculature [24].

Astrocytes also mediate neurotransmission. One of the most widely-studied roles of astrocytes is in the cycling and transport of glutamate, the primary excitatory neurotransmitter in the CNS [25]. Astrocytes and neurons are localized as a tripartite synapse comprised of a presynaptic neuron, postsynaptic neuron, and a neighboring astrocyte [26]. Astrocytes supply neurons with glutamine, which is then converted to glutamate for neurotransmission [12]. After signal transmission, astrocytes reuptake glutamate, convert it to glutamine by astrocytic glutamine synthetase (GS), which is also considered a marker of astrocytes, and export it back to the extracellular space [27].

Astrocytes express numerous potassium (K^+^) and sodium (Na^+^) channels and participate in ionic buffering and the clearance and redistribution of ions from the extracellular space [28]. Extracellular K^+^ induces the depolarization of the astrocytic plasma membrane and leads to bicarbonate-dependent alkalinization, while glutamate uptake causes cytosolic and mitochondrial acidification in astrocytes [28]. Astrocytes also serve as storage sites for glycogen in the CNS and contribute to vasomodulation by releasing molecules such as nitric oxide and arachidonic acid, which mediate cerebral blood flow in response to neuronal activity [29]. Additionally, astrocytes maintain the BBB and regulate fluid flow via expression of the bidirectional water channel aquaporin-4 (AQP4) [30].

As neurons have low levels of endogenous antioxidants, astrocytes also play a critical role in maintaining the CNS antioxidant system and neutralizing ROS/RNS [31]. Astrocytes produce the antioxidants glutathione (GSH) and superoxide dismutase (SODs) and release them into the microenvironment [32]. Astrocytes also release neurotrophic factors such as glial cell line-derived neurotrophic factor (GDNF) and nerve growth factor (NGF), which support neuronal survival [33,34]. In addition to the active secretion of antioxidant and neurotrophic molecules, astrocytes prevent excitotoxicity via clearance of the extracellular space, removing neurotransmitters such as glutamate, gamma-Aminobutyric acid (GABA) and glycine after signal termination [35].

### 2.2. Pathological Role of Reactive Astrocytes

Astrocytes may play a critical role in PD pathogenesis via oxidative and nitrosative stress. Post-mortem analyses of PD patient brains, as well as experimental animal models, indicate that astrocyte activation and elevated levels of ROS/RNS are pathogenic features of PD [36,37]. The activation of astrocytes and oxidative/nitrosative damage are often considered downstream effects of disease, but growing evidence suggests that astrocytic oxidative and nitrosative stress may also contribute to PD [38]. Notably, studies indicate the bidirectional role of astrocytic oxidative/nitrosative stress in both familial and idiopathic PD [39,40,41,42].

Reactive astrogliosis may promote PD pathogenesis. Several studies have demonstrated that astrocytes also receive input from activated microglia, which signal astrocytes through soluble cytokines and chemokines [43]. In times of toxic insult or neuroinflammation, increased release of proinflammatory cytokines such as tumor necrosis factor alpha (TNF-α) and interleukin-1 beta (IL-1β) can induce the morphological and biochemical transformation of astrocytes, known as reactive astrogliosis [44]. Reactive astrogliosis is characterized by changes such as upregulation of GFAP and vimentin, increased expression of astrocytic glutamate transporters and potassium channels, production of antioxidant molecules such as GSH, and the release of ROS/RNS and TNF-α [45].

Although reactive astrocytes contribute to limiting CNS damage during acute injury, prolonged astrogliosis leads to neurotoxicity [46]. The mechanisms by which reactive astrogliosis induces dopaminergic neuronal death are not fully understood, but studies indicate that activated astrocytes produce TNF-α, IL-1β, interleukin-6 (IL-6), and interferon-γ, which initiates neuronal apoptosis through the activation of caspase 3, caspase 8, and cytochrome c [47,48]. Additionally, reactive astrocytes release nitric oxide into the extracellular space, resulting in increased lipid peroxidation, mitochondrial impairment, and DNA strand breaks, which lead to neuronal injury and death [49].

While few studies have examined the role of reactive astrocytes as contributors to PD, current evidence links reactive astrocytes to dopaminergic neurotoxicity [43]. Blocking the activation of astrocytes with NLY01, a glucagon-like peptide-1 receptor (GLP1R) agonist, prevented conversion of astrocytes to the proinflammatory A1 phenotype, attenuating dopaminergic neurodegeneration and motor deficits in a mouse model of sporadic PD [50]. Likewise, reactive astrocytes were found even in early stage PD in experimental macaque models, where they were associated with persistent proinflammatory signaling [51]. Postmortem analysis also indicates the presence of reactive astrocytes in PD patients [7]. Importantly, studies have also demonstrated that α-synuclein released from neurons may be taken up by astrocytes, leading to reactive astrogliosis in an SH-SY5Y-astrocyte co-culture model, as well as in an α-synuclein transgenic mouse model [52]. Taken together, these findings demonstrate that reactive astrogliosis is associated with PD by directly contributing to PD pathogenesis or as a result of its progression.

## 3. Astrocytic Oxidative/Nitrosative Stress: Potential Contributors to PD Pathogenesis

### 3.1. Role of Astrocytic Oxidative Stress in PD Pathogenesis

ROS such as superoxides (O^2−^●), hydroxyl radicals (OH●), and nitric monoxide (NO●) are formed as natural byproducts of oxygen metabolism and play essential roles in cell signaling, gene transcription, and microbial defense [53]. Under physiological conditions, ROS production in the CNS is balanced by the astrocytic antioxidant system, which produces reducing agents which catalyze the breakdown and conversion of ROS to non-reactive products [54]. In addition to the ROS produced via mitochondrial metabolism, the brain also contains high levels of polyunsaturated fatty acids and metals such as copper and iron, which act as reactive substrates and are easily oxidized [55]. When the levels of ROS exceed the detoxifying capacity of the astrocytic antioxidant system, pathological oxidative stress results [56].

Post-mortem analyses of PD patients show elevated levels of oxidative metabolites, the presence of oxidative DNA lesions, and decreased antioxidants in the substantia nigra pars compacta (SNpc) [40,57]. Although the contributions of astrocytic oxidative stress have yet to be elucidated, the presence of oxidative metabolites in PD patients has been correlated with the in vitro aggregation of α-synuclein [57], and the levels of oxidized proteins were found to be significantly higher in the SNpc as compared to the frontal cortex and basal ganglia [39], suggesting that oxidative damage in PD is brain-region specific and may contribute to pathogenesis. The levels of GSH, an antioxidant molecule primarily secreted by astrocytes, are significantly lower in the SNs of PD patients, and the activities of astrocyte-secreted antioxidant enzymes such as SOD, GSH peroxidase (GSH-Px), and catalase (CAT) are markedly decreased in PD patients as compared to healthy controls [58]. Moreover, oxidative DNA lesions are also associated with dopaminergic neuronal loss in patients with Lewy body dementia, suggesting that astrocytic oxidative stress may contribute to PD [39].

Astrocytic oxidative stress may be a consequence of aberrant ROS production induced by mitochondrial dysfunction and/or dopamine (DA) catalysis [59]. ROS are generated as byproducts of metabolism and adenosine triphosphate (ATP) production, processes which occur primarily through oxidative phosphorylation, a series of electron transfers achieved via redox reactions [60]. Dysfunction of mitochondrial complex I, a protein complex along the electron transport chain and the primary site of redox reactions, has been hypothesized to be a primary source of pathogenic ROS and a contributor to PD [61]. Consistent with these suggestions, postmortem analyses showed decreased complex I activity and oxidative damage in sporadic PD patients [62]. Moreover, studies have demonstrated that mitochondrial defects and associated increases in ROS levels impair astrocytic function and lead to toxicity, suggesting that mitochondrial dysfunction may contribute to astrocytic oxidative stress [61].

DA catalysis may also contribute to astrocytic ROS production and oxidative stress [63]. The vesicular transport and storage of DA are decreased in PD brains and in primate models of disease, leading to increased cytosolic DA [64]. Free cytosolic DA is readily oxidized to form quinones or semiquinones, and can result in ROS production that induces reactive astrogliosis [65]. Notably, studies have demonstrated that monoamine-oxidase-mediated DA catabolism generates ROS and results in astrocytic Ca^2+^ cytoplasmic signaling in vitro [66]. Intracellular Ca^2+^ signaling has been established as a key mediator of astrocyte activation, suggesting that neurodegeneration and associated increases in free DA may promote reactive astrogliosis, which contributes to the etiology of PD [67].

### 3.2. Role of Astrocytic Nitrosative Stress in PD Pathogenesis

RNS are generated from the enzymes nitric oxide synthase 2 (NOS) and NADPH oxidase in the healthy brain, where they facilitate diverse functions including vasodilation and neurotransmission [68]. Nitric oxide (NO), a key RNS, mediates signal transduction in the CNS, but under pathological conditions, NO is overproduced and interacts with existing superoxide radicals to produce the RNS peroxynitrite (ONOO^−^) [69]. ONOO^−^ may be further converted to form ROS hydroxyl radicals, leading to oxidative stress and promoting the induction of reactive astrogliosis [70]. Studies have demonstrated that production of ONOO^−^ results in decreased GSH levels in primary rat astrocytes [71], suggesting that aberrant RNS production contributes to disruption of the redox balance in the brain via depletion of astrocytic antioxidant levels, resulting in nitrosative stress.

One mechanism of NO signaling is S-nitrosylation, the covalent attachment of a nitric oxide group to a protein’s cysteine residue [72]. S-nitrosylation, an activating posttranslational modification which is known to induce conformational changes and influence protein interactions and activity, is balanced by enzymatic denitrosylation to maintain the cellular redox balance and prevent nitrosative stress [73]. Under pathological conditions, such as neurodegenerative disease, the high levels of NO produced may lead to aberrant S-nitrosylation, which results in downstream pathological effects, such as protein misfolding and aggregation and disruption of ion homeostasis [74].

Aberrant S-nitrosylation increased the number and permeability of ion channels in rat primary astrocytes [75]. Studies have also demonstrated that NO S-nitrosylates astrocytic glutamate transporters excitatory amino acid transporters 1 and 2 (EAAT1 and EAAT2, respectively) in vivo, reversibly inhibiting glutamate uptake, while mice unable to produce neuronal nitric oxide synthase (nNOS^−/−^) exhibited a decrease in S-nitrosylation and concomitant increases in astrocytic glutamate uptake and glutamate–glutamine cycling as compared to wild-type (WT) mice [76]. Further, the PD-associated protein Parkin is S-nitrosylated in PD patient brains and in mouse models of PD as compared to controls, but the role of astrocytic nitrosative stress in this pathology remains to be elucidated [77].

Astrocytic overproduction of NO also serves as a significant contributor to neuronal mitochondrial impairment [78]. Cytokine stimulation of rat primary astrocyte–neuronal co-culture led to the overproduction of ONOO^−^, resulting in deficiencies in neuronal mitochondrial complexes II, III, and IV which were reversed with the removal of the reactive astrocytes [78]. These findings indicate that generation of RNS and NO-mediated protein modification serve essential roles in the healthy human brain, but pathological conditions such as PD lead to the overproduction of RNS and aberrant S-nitrosylation that contribute to further pathology.

## 4. Neuroinflammation

The majority of PD cases are of unknown etiology, but numerous studies demonstrate that neuroinflammatory signaling significantly contributes to the initiation of reactive astrogliosis and consequent astrocytic oxidative/nitrosative stress [79,80,81]. Experimental evidence also indicates that neuroinflammation and astrocytic oxidative/nitrosative stress reciprocally modulate each other to promote neurotoxicity and PD pathogenesis [82].

Under pathological conditions, inflammatory stimuli, such as IL-1β, lipopolysaccharides (LPS), and TNF-α, induce reactive astrogliosis in vitro and in animal models of PD [44]. Activation of microglia via LPS induced the secretion of TNF-α, IL-1, and complement component 1, subcomponent q (C1q), which resulted in the transformation of purified mouse astrocytes towards a classically reactive A1 phenotype [43]. This finding was paralleled in vivo, where LPS injection led to rapid increase in the number of reactive astrocytes and astrocytic production of proinflammatory cytokines in WT mice, but not TNF-α receptor-null (TNF R1/R2(−/−)) mice, suggesting that chronic inflammation contributes to astrocytic reactivity and pathogenesis [83].

Activation of nuclear factor kappa-B (NF-κB), an essential mediator of neuroinflammation, plays an important role in the initiation of reactive astrogliosis and PD pathogenesis [84]. Expression of the NF-κB p65 subunit is significantly higher in the midbrain of PD patients as compared to age-matched controls, and immunofluorescent analysis indicates that exposure to inflammatory stimuli induces robust increases in astrocytic expression of p65 in an MPTP mouse model [85]. The upregulation of NF-κB initiates marked increase in astrocytic ROS/RNS generation and proinflammatory cytokine release, initiating a toxic feedforward loop of chronic inflammation and astrocytic oxidative/nitrosative stress, leading to neurotoxicity [86].

## 5. PD-Associated Genes and Astrocytic Oxidative/Nitrosative Stress

**Figure 2:**
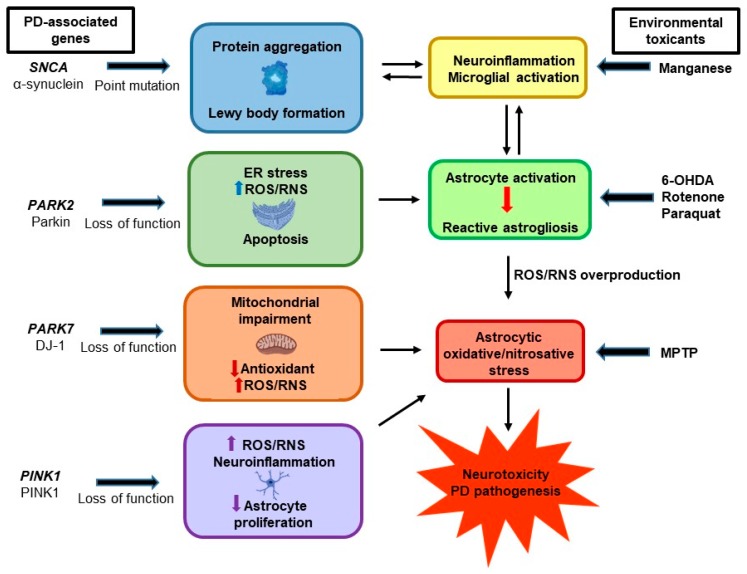
Gene–environment interactions contributing to astrocytic oxidative and nitrosative stress and Parkinson’s disease (PD) pathogenesis. Aberrant functions of PD-associated genes, such as SNCA, PARK2, PARK7, and PINK1, induce astrocytic oxidative/nitrosative stress. In addition, exposure to environmental factors such as 1-methyl-4-phenyl-1,2,3,6-tetrahydropyridine (MPTP), 6-hydroxydopamine (6-OHDA), rotenone, manganese, and paraquat, are also associated with astrocytic oxidative/nitrosative stress and PD.

While the majority of PD cases are idiopathic in nature, studies have identified mutations in several genes that are associated with familial and sporadic PD [87]. However, growing evidence reveals that genes such as α-synuclein, DJ-1, Parkin, and PINK1 may contribute to astrocytic oxidative/nitrosative stress and promote pathogenesis—or protect against it [13] (Figure 2).

### 5.1. α-Synuclein

As the primary component of Lewy bodies, α-synuclein is closely associated with PD [88]. Encoded by the *SNCA* gene, duplication, triplication, and point mutations were among the first gene variants identified as causative factors in early-onset familial PD [89]. Under physiological conditions, α-synuclein is localized primarily in the presynaptic nerve terminals and serves to facilitate vesicular trafficking and SNARE complex formation, but pathological changes to α-synuclein lead to its aggregation and fibrillation, resulting in the formation of Lewy bodies [90].

While the mechanisms of toxicity are not well understood, accumulated α-synuclein was found in the cytoplasm of protoplasmic astrocytes in nearly half of all analyzed PD patient brains [91]. Additionally, co-culture of primary astrocytes with SH-SY5Y human neuroblastoma cells secreting α-synuclein resulted in the formation of astrocytic Lewy bodies [92], suggesting that neuronal α-synuclein may possess prion-like activity which results in astrocytic dysfunction. As the endogenous expression of *SNCA* is low in astrocytes [93], these findings suggest that the pathological aggregation of α-synuclein contributes to astrocytic dysfunction and oxidative/nitrosative stress.

The astrocytic accumulation of α-synuclein was associated with increased production of proinflammatory cytokines such as IL-1, IL-6, and TNF-α, as well as the release of chemokines such as C-X-C motif ligand 1 (CXCL1) in vitro [83]. Studies also indicate that elevated levels of extracellular α-synuclein induce a concentration-dependent inflammatory response in primary astrocyte cultures [84]. Accordingly, A53T mice overexpressing mutant α-synuclein displayed a significant increase in reactive astrocytes and increased production of ROS and proinflammatory prostaglandins such as cyclooxygenase 1 (COX-1) as compared to WT, suggesting that accumulation of α-synuclein leads to neuroinflammation, reactive astrogliosis, and consequent PD pathogenesis [94]. While further studies are needed to elucidate the mechanisms of α-synuclein toxicity, these findings demonstrate the significant impact of α-synuclein aggregation on astrocytic dysfunction in PD.

### 5.2. DJ-1

Protein deglycase DJ-1 (DJ-1), encoded by the *PARK7* gene, assists in protein folding and downstream proteosomal localization, and its mutation is associated with the aggregation of α-synuclein and autosomal recessive PD [95]. Notably, studies have indicated that DJ-1 confers protection to astrocytes against oxidative/nitrosative stress [96]. DJ-1 is upregulated in reactive astrocytes in postmortem analyses of sporadic PD patients, and in vitro studies indicate that DJ-1 upregulation is associated with increased astrocytic release of soluble antioxidant molecules in a primary astrocyte–neuron co-culture model [97,98]. Moreover, overexpression of DJ-1 in astrocytes protected co-cultured neurons against oxidative stress in a rotenone mouse model, while DJ-1 knockout (DJ-1 KO) decreased the neuroprotective capacity of astrocytes and resulted in decreased neuronal survival [99]. Taken together, these findings suggest that increased DJ-1 expression serves as a compensatory mechanism to overcome oxidative stress.

In contrast to genes which promote astrocytic defects, DJ-1 may prevent the production of ROS/RNS in astrocytes [100]. In a neuron–astrocyte co-culture model, astrocytes with DJ-1 deletion produced greater than ten (10) times more NO than their WT counterparts [101]. DJ-1 mutation also resulted in the sustained astrocytic release of proinflammatory molecules IL-6 and cyclooxygenase-2 (COX-2) and subsequent neuronal death, suggesting a significant role for DJ-1 in preventing astrocytic nitrosative stress and inflammation in PD pathogenesis [102]. *PARK7* overexpression may protect astrocytic function and prevent oxidative stress via promotion of astrocytic antioxidant production [103]. Rats overexpressing astrocytic DJ-1 exhibited significant reductions in rotenone-induced oxidative stress, microglial activation, and dopaminergic neuronal loss as compared to the WT, suggesting that DJ-1 confers significant protection to astrocytes and prevents astrocytic oxidative stress [104]. Astrocytic DJ-1 overexpression also prevented mitochondrial complex I inhibition and oxidative stress in a transgenic zebrafish model [100]. Further, astrocytic DJ-1 overexpression resulted in the upregulation of proteins associated with redox regulation, prevented NO production, and inhibited protein nitrosylation, suggesting that DJ-1 mediates astrocytic function and prevents astrocytic oxidative/nitrosative stress [100].

### 5.3. Parkin

Parkin, a ubiquitin E3 ligase encoded by the *PARK2* gene, contributes to mitochondrial integrity and regulates the mitophagic degradation of proteins under physiological conditions [105]. While *PARK2* mutations are the most common genetic defects found in early onset familial PD, evidence also suggests that Parkin may play an astrocyte-specific role in PD pathogenesis [106]. Astrocytes highly express Parkin at times of intracellular stress [107], and Parkin-KO primary astrocytes exhibit significant increase in nucleotide-oligomerization domain receptor 2 (NOD2), a cytosolic receptor which initiates inflammatory signaling and ROS/RNS production in response to endoplasmic reticulum (ER) stress, as compared to WT astrocytes [108,109]. Parkin-KO astrocytes also displayed increased ER stress and cytokine production, as well as decreased astrocytic secretion of antioxidant molecules such as brain-derived neurotrophic factor (BDNF) and GDNF [109]. Consistent with these results, cinnamon treatment upregulated Parkin and DJ-1 in astrocytes, leading to decreased ER stress and reduction of ROS/RNS and proinflammatory cytokines [110]. Astrocytic Parkin defects may also contribute to neurotoxicity through dysregulation of the cellular redox balance and induction of apoptosis [111]. Astrocytes isolated from Parkin-KO mice exhibited decreased astrocyte proliferation and increased proapoptotic protein expression [112]. As ER stress and apoptosis are one of the primary mechanisms of neurodegeneration [108], Parkin may play a critical role in preserving astrocytic function and preventing oxidative/nitrosative stress.

### 5.4. PINK1

PTEN-induced putative kinase 1 (PINK1) is a serine/threonine kinase of which the mutation is associated with early-onset autosomal recessive PD [12]. Encoded by the *PINK1* gene, PINK1 contributes to astrocyte development and proliferation, facilitates the autophagic degradation of damaged mitochondria, and may play a protective role in astrocytic oxidative/nitrosative stress and PD [113]. Accordingly, PINK1-KO astrocytes exhibit significant increases in NO, TNF-α, and IL-1β production and enhanced NF-κB activation under neuroinflammatory conditions as compared to WT astrocytes [114]. Moreover, PINK1-KO mice exhibited decreased astrocyte proliferation and increased astrocytic ROS levels as compared to WT mice, suggesting that aberrant PINK1 contributes to astrocytic dysfunction via increased production of ROS/RNS and decreases in astrocytic proliferation [115].

## 6. Experimental Models and Astrocytic Oxidative/Nitrosative Stress

As only ~10% of PD cases can be attributed to genetic causes, the role of environmental factors in astrocytic oxidative/nitrosative stress and PD pathogenesis has emerged as an area of focus. Herbicides, pesticides, fungicides, and other toxicants, including MPTP, rotenone, and paraquat, have been utilized in cellular and animal models to delineate the mechanisms of pathogenesis [116]. Exposure to environmental toxicants induces astrocytic toxicity: the production of ROS/RNS, decrease in endogenous antioxidant levels, induction of reactive astrogliosis, and astrocytic cell death [34,116], although the mechanisms of toxicity remain to be fully elucidated.

### 6.1. MPTP

MPTP, a widely utilized Parkinsonian neurotoxicant, is a lipophilic compound which rapidly crosses the blood–brain barrier and undergoes uptake by nigrostriatal astrocytes, where it is metabolized to the toxic byproduct 1-methyl-4-phenylpyridinium (MPP^+^) [117]. MPP^+^ is then transported into presynaptic dopaminergic nerve terminals through the action of dopamine transporters [118]. Although astrocytic dopamine transport occurs to a lesser extent [119], studies have demonstrated that overexpression of dopamine transporter increased sensitivity and vulnerability of mice to MPTP-induced neurotoxicity, indicating an important role for dopamine transporters in PD pathology [120]. While studies of MPTP-induced toxicity have primarily focused upon the mechanisms of MPTP transport and dopaminergic neurotoxicity, reactive astrocytes may serve as key contributors to MPTP-induced pathogenesis through the production of astrocytic ROS/RNS and loss of function [121]. Exposure to MPTP significantly increased the production of ROS and decreased the levels of the antioxidant molecule SOD in primary mouse astrocyte cultures [122]. MPTP-treated mice also exhibited the upregulation of proinflammatory cytokines such as IL-1β and TNF-α, and the associated astrocytic release of ROS and NO as compared to controls [123]. Likewise, studies found that MPTP treatment resulted in increased levels of astrocytic myeloperoxidase (MPO), a lysosomal enzyme which can induce the release of proinflammatory cytokines, astrocytic production of ROS/RNS, and oxidative DNA damage in the SN in a mouse model, suggesting that astrocytic oxidative/nitrosative stress significantly contributes to MPTP toxicity [124].

### 6.2. Rotenone

Rotenone is a neurotoxic pesticide and insecticide which crosses the BBB, inducing PD-like pathology in experimental models [125]. Rotenone competitively inhibits mitochondrial complex I, impairing mitochondrial function and promoting oxidative/nitrosative stress in C6 astrocytes [126]. Rotenone exposure increased the levels of astrocytic NO and MPO in a primary neuron–astrocyte co-culture model, suggesting that rotenone may exert toxic effects in part via astrocytic oxidative/nitrosative stress [127]. Additionally, rotenone induced increased astrocyte proliferation and morphological changes in astrocytes in the SN of rats, suggesting that reactive astrogliosis may serve a mechanism of rotenone toxicity and associated pathogenesis [128]. Rotenone also induced ROS production and decreased antioxidant levels in cultured astrocytes, leading to the impairment of mitochondrial membrane potential and astrocytic toxicity in T98G human astrocytes [129], indicating that astrocytic oxidative/nitrosative stress plays a critical role in rotenone-induced toxicity.

### 6.3. Mn

Chronic exposure to Mn induces a neurological disorder referred to as manganism, which shares similar features with PD, such as dopaminergic neurodegeneration and motor deficits [130]. While the mechanisms of Mn-induced neurotoxicity are not fully understood, astrocytic oxidative/nitrosative stress may play a significant role in Mn pathogenesis [131]. Mn increased ROS levels and NO production in C6 astrocytes in an NF-κB-dependent manner, suggesting that neuroinflammation contributes to pathological astrocytic oxidative/nitrosative stress in Mn toxicity [132]. Mn exposure also induces the production of proinflammatory cytokines such as TNF-α, IL-6, and IL-1β, prostaglandins, and NO in astrocytes, disrupts the activity of enzymes such as glutamine synthetase, and decreases the expression and activity of astrocytic glutamate transporters, leading to excitotoxicity [133,134]. In contrast, Mn-induced toxicity is reversed with treatment with antioxidants and increased GSH levels in rat astrocytes, providing further evidence for the role of astrocytic oxidative/nitrosative stress in Mn-induced astrocytic injury [135]. Moreover, Mn increases the astrocytic expression of inducible nitric oxide synthase (iNOS), an enzyme which catalyzes the production of NO, in a mouse model, while neurotoxicity was attenuated with the genetic deletion of iNOS [136,137]. Studies also indicate that treatment with 17β-estradiol (E2) and tamoxifen (TX) attenuates ROS production and reverse oxidative stress in primary astrocytes and in mice [138,139]. Taken together, these findings indicate that Mn-induced neurotoxicity involves astrocytic oxidative/nitrosative stress.

### 6.4. Paraquat

Paraquat (N,N′-dimethyl-4,4′-bipyridinium dichloride) is an herbicide which induces dopaminergic lesions and neuronal cell death in animal models [125]. However, evidence suggests that paraquat also promotes astrocytic oxidative/nitrosative stress. Paraquat induces rapid increases in intracellular antioxidant levels and enhances GFAP expression in cultured human and rat astrocytes, suggesting that paraquat exposure initiates reactive astrogliosis to maintain the cellular redox balance [140,141]. Paraquat also induces the upregulation of genes associated with oxidative stress, redox maintenance, and apoptosis, including heme oxygenase-1, NAD(P)H dehydrogenase, and glutathione S-transferase P, in primary astrocytes [142]. Moreover, paraquat impairs autophagic function in U-373 MG astrocytes, leading to decreased antioxidant capabilities and toxicity [143]. These findings suggest that astrocytic oxidative/nitrosative stress plays a significant role in paraquat-induced neurotoxicity.

#### 6.5. 6-OHDA

6-Hydroxydopamine (6-OHDA) is a neurotoxin which induces Parkinsonian motor deficits and dopaminergic neurodegeneration in experimental models [144]. Several studies found that 6-OHDA induced the activation of astrocytes in the SN of rats, leading to the upregulation of GFAP and morphological changes associated with reactive astrogliosis [145,146]. Pharmacological inhibition of the inflammatory receptor for advanced glycation end product (RAGE) blocked the activation of astrocytes and attenuated dopaminergic neurodegeneration in 6-OHDA-injected rats, suggesting that the initiation of reactive astrogliosis serves as an initiating mechanism of 6-OHDA-induced neurotoxicity [147]. 6-OHDA increased ROS production and decreased the levels of the antioxidant molecules SOD and CAT in astrocytes, which was attenuated by treatment with octadecaneuropeptide, an endogenous benzodiazepine receptor ligand [148]. The secretion of astrocytic GDNF and GSH also protected neurons from 6-OHDA-induced toxicity in mouse and human astrocyte–neuron co-culture models [149], suggesting that astrocytic oxidative/nitrosative stress serves as a mechanism of 6-OHDA toxicity.

## 7. Pharmacological Attenuation of Astrocytic Oxidative/Nitrosative Stress in PD

While reactive astrocytes have emerged as an important contributor to pathogenesis [12,150], few studies have examined therapeutic approaches to attenuating astrocytic oxidative/nitrosative stress in PD. Salidroside, extracted from *Rhodiola rosea*, decreased ROS production and increased viability in a neuron–astrocyte co-culture model [151]. Likewise, treatment with coenzyme Q10 (CoQ10) decreased ROS production in cultured astrocytes and improved viability [152]. Clinical trials of CoQ10 have also shown modest therapeutic benefit, suggesting that targeting astrocytic oxidative/nitrosative stress may constitute a new therapeutic approach to PD [153]. Treatment with synthetic triterpenoids, which increase activation of the cytoprotective transcription factor nuclear factor E2-related factor 2 (Nrf2), have also shown therapeutic promise in an MPTP mouse model; decreasing dopaminergic neurotoxicity [154]. Together, these findings indicate a continued need for exploration of the therapeutic attenuation of PD via the targeting of astrocytic oxidative/nitrosative stress.

## 8. Conclusions

Astrocytic oxidative/nitrosative stress has been strongly linked to PD pathogenesis. While astrocytes provide structural and metabolic support to neurons, play a critical role in maintaining redox balance, and regulate the antioxidant system in the CNS, aberrant reactive astrogliosis leads to the chronic production of ROS/RNS and contributes to PD progression. Accordingly, delineating the molecular mechanisms involved in astrocytic oxidative/nitrosative stress is critical to furthering our understanding of astrocyte dysfunction and its role in neuropathogenesis. PD-related genes such as α-synuclein, DJ-1, Parkin, and PINK1 mediate astrocytic function and significantly impact astrocytic oxidative/nitrosative stress. Experimental models utilizing environmental toxicants such as MPTP, rotenone, manganese, paraquat, and 6-OHDA also provide valuable insight into the mechanisms of astrocytic oxidative/nitrosative stress and their impact on pathogenesis. Altogether, the findings presented in this review provide evidence for the impact of astrocytic oxidative/nitrosative stress on PD pathogenesis. Further studies elucidating the role of astrocytic oxidative/nitrosative stress will enhance understanding of the mechanisms of pathogenesis and facilitate the identification of novel therapeutic approaches to treating PD.
